# Coverage and Access Changes During Medicaid Unwinding

**DOI:** 10.1001/jamahealthforum.2024.2193

**Published:** 2024-06-29

**Authors:** Adrianna McIntyre, Benjamin D. Sommers, Gabriella Aboulafia, Jessica Phelan, E. John Orav, Arnold M. Epstein, Jose F. Figueroa

**Affiliations:** 1 Harvard University T.H. Chan School of Public Health, Department of Health Policy and Management, Boston, Massachusetts; 2 Harvard Medical School, Boston, Massachusetts; 3 Brigham and Women’s Hospital, Department of Medicine, Boston, Massachusetts

## Abstract

**Importance:**

States resumed Medicaid eligibility redeterminations, which had been paused during the COVID-19 public health emergency, in 2023. This unwinding of the pandemic continuous coverage provision raised concerns about the extent to which beneficiaries would lose Medicaid coverage and how that would affect access to care.

**Objective:**

To assess early changes in insurance and access to care during Medicaid unwinding among individuals with low incomes in 4 Southern states.

**Design, Setting, and Participants:**

This multimodal survey was conducted in Arkansas, Kentucky, Louisiana, and Texas from September to November 2023, used random-digit dialing and probabilistic address-based sampling, and included US citizens aged 19 to 64 years reporting 2022 incomes at or less than 138% of the federal poverty level.

**Exposure:**

Medicaid enrollment at any point since March 2020, when continuous coverage began.

**Main Outcomes and Measures:**

Self-reported disenrollment from Medicaid, insurance at the time of interview, and self-reported access to care. Using multivariate logistic regression, factors associated with Medicaid loss were evaluated. Access and affordability of care among respondents who exited Medicaid vs those who remained enrolled were compared, after multivariate adjustment.

**Results:**

The sample contained 2210 adults (1282 women [58.0%]; 505 Black non-Hispanic individuals [22.9%], 393 Hispanic individuals [17.8%], and 1133 White non-Hispanic individuals [51.3%]) with 2022 household incomes less than 138% of the federal poverty line. On a survey-weighted basis, 1564 (70.8%) reported that they and/or a dependent child of theirs had Medicaid at some point since March 2020. Among adult respondents who had Medicaid, 179 (12.5%) were no longer enrolled in Medicaid at the time of the survey, with state estimates ranging from 7.0% (n = 19) in Kentucky to 16.2% (n = 82) in Arkansas. Fewer children who had Medicaid lost coverage (42 [5.4%]). Among adult respondents who left Medicaid since 2020 and reported coverage status at time of interview, 47.8% (n = 80) were uninsured, 27.0% (n = 45) had employer-sponsored insurance, and the remainder had other coverage as of fall 2023. Disenrollment was higher among younger adults, employed individuals, and rural residents but lower among non-Hispanic Black respondents (compared with non-Hispanic White respondents) and among those receiving Supplemental Nutrition Assistance Program benefits. Losing Medicaid was significantly associated with delaying care due to cost and worsening affordability of care.

**Conclusions and Relevance:**

The results of this survey study indicated that 6 months into unwinding, 1 in 8 Medicaid beneficiaries reported exiting the program, with wide state variation. Roughly half who lost Medicaid coverage became uninsured. Among those moving to new coverage, many experienced coverage gaps. Adults exiting Medicaid reported more challenges accessing care than respondents who remained enrolled.

## Key Points

**Question:**

How did insurance coverage and access to care change among low-income households during the Medicaid unwinding process?

**Findings:**

In this survey study of US low-income households in 4 Southern states in late 2023, 1 in 8 respondents who had Medicaid reported exiting the program roughly 6 months into the unwinding process, with wide state-level variation in coverage loss. Roughly half of those in the sample who lost Medicaid coverage became uninsured.

**Meaning:**

The results of this study suggest that state and federal policymakers should pursue policies to mitigate adverse outcomes associated with coverage disruptions during and after Medicaid unwinding.

## Teaser

This survey study examines assess early changes in insurance and access to care during Medicaid unwinding among individuals with low incomes in 4 Southern states.

## Introduction

As part of the COVID-19 federal public health emergency, states paused Medicaid disenrollment in exchange for increased federal funding, allowing Medicaid beneficiaries to remain continuously enrolled without eligibility redeterminations.^1^ This policy was associated with record historic growth in Medicaid and the Children’s Health Insurance Program (CHIP), increasing from roughly 72 million in March 2020 to more than 92 million people by December 2022.^2,3,4,5,6^ In late 2022, Congress passed legislation to end the continuous coverage provision; states resumed eligibility redeterminations in early and mid 2023.^7^

Initial projections suggested that this unwinding of continuous coverage would be followed by 15 to 18 million people losing Medicaid benefits.^8^ However, by early May 2024, the number of people disenrolled from Medicaid exceeded 21 million.^9^ Most people who have lost Medicaid thus far (70%) were disenrolled due to administrative or procedural reasons, which include the inability or failure to complete paperwork, rather than confirmed loss of eligibility.^9^ Policies governing this process and disenrollment rates vary considerably by state.^10,11^

While administrative data show the number of enrollees losing Medicaid coverage, they do not track enrollees’ coverage transitions nor offer insights into how unwinding is associated with enrollees’ access to and affordability of medical care.^12^ High-quality federal surveys will eventually illuminate some of these dynamics, but these data are subject to considerable time lag.^13^

To provide timely insights into how Medicaid unwinding affects individuals in the US experiencing low income, we conducted a multimodal survey of adults in 4 states during late 2023. Respondents were US citizens who reported 2022 incomes less than 138% of the federal poverty line (FPL). The survey assessed changes in insurance coverage and access to care.

The 4 states in our sample (Arkansas, Kentucky, Louisiana, and Texas) took varied approaches to unwinding. Arkansas conducted redeterminations on an accelerated 6-month timeline; most states took a year.^14^ Kentucky and Louisiana spread redeterminations evenly across 12-month schedules; however, Kentucky halted redeterminations for children for a year, extending their continuous coverage while unwinding was underway for adults.^15,16,17^ Texas conducted redeterminations over a full year, prioritizing cases thought likely to be ineligible, and aiming to conduct most redeterminations during the first 6 months.^18^ The federal government allowed states to waive certain requirements to implement strategies to improve the retention of eligible enrollees during unwinding; the number of these optional strategies pursued by states in the sample ranged from 4 (Texas) to 14 (Kentucky).^49^ Additional details on the unwinding policies of states are available in Supplement 1.

## Methods

### Study Design, Setting, and Sample

We conducted a representative survey of US citizens with low incomes in 4 Southern states (Arkansas, Kentucky, Louisiana, and Texas) between September 18, 2023, and November 21, 2023. The survey primarily recruited respondents through random-digit dialing (using cellular and landline telephones) and probabilistic address-based sampling (ABS). Respondents recruited through ABS received postcards inviting them to participate in the survey by phone or on the internet. A small proportion of the sample was recruited using the survey vendor’s nationally representative probability-based web panel or by contacting individuals probabilistically recruited for prior unrelated research. Informed consent was obtained directly through the online survey format or verbally for those participating by phone. The study followed American Association for Public Opinion Research (AAPOR) reporting guidelines and was approved by the Harvard T.H. Chan School of Public Health institutional review board.

The sample contained US citizens aged 19 to 64 years who reported family incomes in 2022 less than 138% of the FPL. This income criterion reflects the eligibility threshold for Medicaid in states that have expanded the program under the Affordable Care Act. The survey oversampled respondents who self-identified as Black or Hispanic to facilitate investigation of potential racial disparities. We also oversampled Texas (the lone nonexpansion state) and Arkansas (the first of these states to resume Medicaid redeterminations in 2023).

This study was a continuation of repeated cross-sectional surveys in these states, and previous research demonstrated that this survey approach has produced state-level coverage trends that closely track with subsequent data from the US Census Bureau.^19^ Additional information about survey design is in Supplement 1.

The survey collected information on demographic characteristics (including self-reported race and ethnicity), current health insurance, and access to care. We also asked respondents whether they had been enrolled in Medicaid at any point since March 2020, when continuous coverage began. Respondents with dependent children (younger than 19 years) were asked about their child’s insurance at the time of the interview and whether the child had any Medicaid/CHIP coverage since March 2020. Survey items were primarily drawn from prior versions of this survey, which adapted from federal government surveys or recent survey questions used by KFF and Urban Institute.^20,21,22^

### Outcomes

Among respondents (and, when applicable, their children) who had Medicaid coverage at any point since March 2020, the primary outcome was self-reported disenrollment from Medicaid (that is, not reporting Medicaid coverage at the time of the interview). Secondary outcomes were current health care coverage among Medicaid disenrollees (Medicare, employer-sponsored insurance, marketplace insurance, other coverage, or uninsured), whether respondents had experienced a gap in coverage (lasting 1 month or longer) during the previous year, and several measures of access to and affordability of care: delayed care during the previous year due to cost, delayed medications during the previous year due to cost, reporting care was less affordable than a year ago, and whether a person had a checkup during the previous year. Exact survey question wording is available in the eMethods in Supplement 1.

### Statistical Analysis

First, we summarized characteristics of the full sample and subset of respondents who reported having Medicaid coverage since March 2020. We then estimated rates for the primary outcome, loss of Medicaid, stratifying by state for adult respondents. To assess the validity of our results, we compared state-level Medicaid losses reported in the sample with Medicaid disenrollment rates in administrative data that were concurrent with the timing of our survey and estimated the correlation coefficient for those estimates. We then evaluated insurance at the time of the survey among adult respondents reporting Medicaid disenrollment and whether respondents had a gap in coverage during the previous year.

Using multivariate logistic regression, we separately examined factors associated with Medicaid loss among adults and children (for children, we excluded Kentucky from this model, since it did not disenroll any children in 2023). The covariates were state of residence; demographic characteristics, including race and ethnicity, age, education, employment, income, and parental status (for adults); receipt of Supplemental Security Income (SSI), receipt of Supplemental Nutritional Assistance Program (SNAP) benefits (which may be associated with an increased likelihood that a person had been in contact with state agencies or that the state had adequate income information for their eligibility redetermination); and whether the respondent had moved since March 2020 (which may have been associated with a reduced likelihood that a person received renewal paperwork).^23,24^

We then used a multivariate logistic regression (adjusting for the previously described covariates) to compare access and affordability measures among respondents who exited Medicaid vs those who remained enrolled in the program. All analyses were survey weighted using state-specific benchmarks derived from federal data for the demographic variables listed in the previously described models; each state was weighted in proportion to its share of the sample (ie, more populous states were not weighted more heavily). Weights also adjusted for modality and nonresponse. Statistical analyses were conducted using Stata, version 17 (StataCorp); significance was determined at the 5% level.

## Results

### Study Sample and Descriptive Statistics

The survey sample comprised 2210 respondents; 1471 (66.6%) reported Medicaid enrollment since March 2020 themselves, and 766 (34.7%) reported child Medicaid enrollment (636 respondents reported Medicaid for themselves and a child). A total of 1155 respondents (52%) were recruited through ABS and 930 (42%) through random-digit dialing; the rest were recruited from the vendor’s prior surveys (85 [4%]) or a probability-based web panel (40 [2%]). The overall response rate was 5%.

A total of 1282 participants (35.8%) resided in Texas and 728 (32.9%) in Arkansas, with the remainder split between Kentucky (351 [15.9%]) and Louisiana (791 [15.4%]). Before weighting, 27.8% (n = 616) of the sample self-identified as non-Hispanic Black, 18.2% (n = 402) as Hispanic, 46.5% (n = 1028) as non-Hispanic White, and 7.4% (n = 164) as another race (including Asian, American Indian or Alaskan Native, and Hawaiian or other Pacific Islander).

After applying survey weights, 1564 (70.8%) reported that either they and/or a dependent child had been enrolled in Medicaid at some point since March 2020. Table 1 presents summary statistics for the full study sample and the subset of the sample reporting Medicaid enrollment since March 2020.

**Table 1.** Characteristics of the Study Sample^a^

Characteristic	No. (%)		
	Respondent and/or child had Medicaid at any time since March 2020 (unweighted, n = 1601; weighted, n = 1564)	Respondent and/or child did not have Medicaid at any time since March 2020 (unweighted, n = 609; weighted, n = 646)	Full sample (N = 2210)
State			
Arkansas	557 (35.7)	171 (26.4)	728 (32.9)
Kentucky	282 (18.0)	69 (10.7)	351 (15.9)
Louisiana	297 (19.0)	43 (6.7)	340 (15.4)
Texas	428 (27.4)	363 (56.2)	791 (35.8)
Women	1000 (64.0)	281 (43.5)	1282 (58.0)
Age, y			
19-29	436 (27.9)	269 (41.6)	705 (31.9)
30-39	378 (24.2)	109 (16.8)	487 (22.0)
40-49	276 (17.6)	89 (13.8)	365 (16.5)
50-59	298 (19.0)	104 (16.1)	402 (18.2)
60-64	176 (11.3)	75 (11.6)	252 (11.4)
Race and ethnicity			
Black non-Hispanic	377 (24.1)	128 (19.9)	505 (22.9)
Hispanic	244 (15.6)	149 (23.0)	393 (17.8)
White non-Hispanic	825 (52.7)	308 (47.7)	1133 (51.3)
Other^b^	118 (7.6)	61 (9.5)	180 (8.1)
Education level			
Less than high school degree	276 (17.7)	95 (14.8)	372 (16.8)
High school graduate	685 (43.8)	233 (36.0)	918 (41.5)
Some college/college graduate	602 (38.5)	318 (49.3)	921 (41.7)
Family income			
Less than 50% FPL	600 (38.4)	188 (29.1)	788 (35.7)
50%-100% FPL	609 (38.9)	208 (32.2)	817 (37.0)
100%-138% FPL	317 (20.2)	230 (35.6)	547 (24.8)
Do not know/refused	38 (2.4)	20 (3.1)	58 (2.6)
Currently employed	615 (39.4)	361 (55.9)	976 (44.3)
Married or living with a partner	609 (38.9)	263 (40.7)	872 (39.5)
Has dependent child	1115 (71.3)	283.4 (44.0)	1398 (63.3)
Rural	536 (34.3)	134 (20.8)	670 (30.3)
Has chronic condition	1212 (77.5)	434 (67.1)	1646 (74.5)
Continuity of coverage			
Insured all year	1166 (75.2)	332 (52.3)	1498 (68.5)
Had coverage gap	337 (21.7)	123 (19.3)	460 (21.0)
Uninsured all year	48 (3.1)	180 (28.4)	228 (10.4)
No longer has Medicaid coverage	179 (12.5)	NA	NA
Benefits receipt			
SSI	459 (29.4)	60 (9.4)	519 (23.6)
SNAP	856 (55.1)	108 (16.9)	964 (44.0)
Moved since March 2020	702 (44.9)	323 (50.1)	1025 (46.4)

Abbreviations: FPL, federal poverty line; NA, not applicable; SNAP, Supplemental Nutrition Assistance Program; SSI, supplemental security income.

^a^ Data are from a multimodal (telephone + internet) survey of nonelderly US citizens (aged 19-64 years) who lived in 1 of 4 Southern states (Arkansas, Kentucky, Louisiana, and Texas) and reported 2022 household incomes less than 138% of the FPL. For the gender variable, respondents were able to select man, woman, or another identity; respondents from the last group were not shown due to small sample size. Respondents who are categorized as “do not know/refused” for the income variable attested to having 2022 household income less than 138% FPL but did not provide additional details on their income. Respondents were asked if they had any of the following chronic conditions: high blood pressure; a heart attack, coronary artery disease, or heart failure; asthma, chronic bronchitis, chronic obstructive pulmonary disease, or emphysema; diabetes; depression or anxiety; cancer, except for skin cancer; or alcoholism or drug addiction. The survey was fielded from September to November 2023. Percentages/counts may not sum as expected due to rounding. All reported counts and proportions (aside from sample sizes) are survey-weighted. The weighted N in Table 1 adjusts for the proportional population share across the 4 states.

^b^ The “Other” category for the race and ethnicity variable included those who identify as Asian, American Indian or Alaskan Native, Native Hawaiian or other Pacific Islander, or who selected the other response in the survey. Respondents are not broken out due to small sample size. Race and ethnicity were self-reported.

### Disenrollment From Medicaid and Subsequent Insurance Coverage

**Figure 1.**
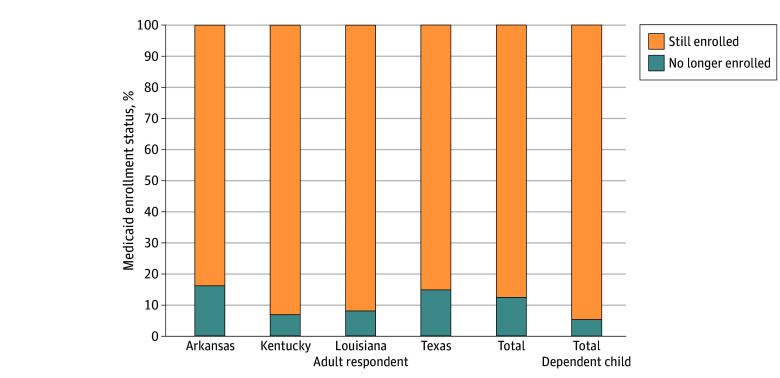
Medicaid Enrollment Status at the Time of Interview Among Respondents Ever in Medicaid Since March 2020 Data were from a multimodal (telephone + internet) survey of nonelderly US citizens (aged 19-64 years) who lived in 1 of 4 Southern states (Arkansas, Kentucky, Louisiana, and Texas) and reported 2022 household incomes less than 138% of the federal poverty line and that they and/or a dependent child (if any) had been enrolled in Medicaid at some point since March 2020. The survey was fielded from September to November 2023. Percentages may not sum to 100 due to rounding. All reported estimates were survey-weighted.

Overall, 12.5% (n = 179) of adults who had Medicaid at some point since March 2020 were no longer enrolled by fall 2023, ranging from 7.0% (n = 19) in Kentucky to 16.2% (n = 82) in Arkansas (Figure 1), with Louisiana (23 [8.2%]) and Texas (54 [14.9%]) falling in between. Fewer dependent children (42 [5.4%] overall) lost Medicaid coverage. The state-level estimates of adult coverage loss were strongly correlated with administrative records of coverage loss in late 2023 (ρ = 0.92); additional details are available in the eAppendix in Supplement 1.

**Figure 2.**
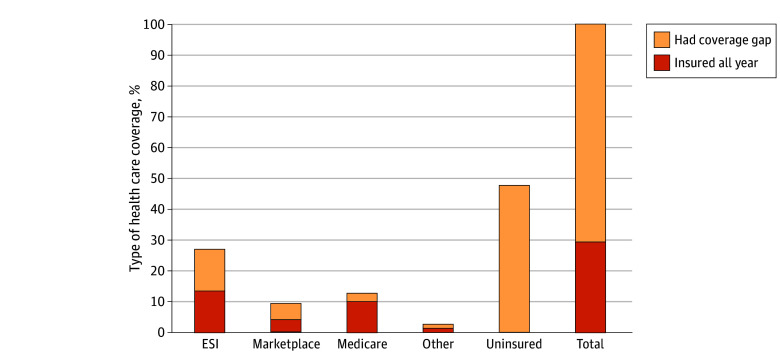
Type of Health Care Coverage Among Adults Who Disenrolled From Medicaid Survey-weighted rates of coverage at the time of the interview among people who reported disenrolling from Medicaid. Data were from a multimodal (telephone + internet) survey of nonelderly US citizens (aged 19-64 years) who lived in 1 of 4 Southern states (Arkansas, Kentucky, Louisiana, and Texas) and reported 2022 household incomes less than 138% of the federal poverty line and reported that they had been enrolled in Medicaid at some point since March 2020. The survey was fielded from September to November 2023. Percentages may not sum to 100 due to rounding. All reported estimates were survey-weighted. ESI indicates employer-sponsored insurance.

Among adults who lost Medicaid coverage (n = 168), just fewer than half (80 [47.8%]) were uninsured at the time of the interview (Figure 2), while 52.2% had other coverage. Among disenrollees, 27% (n = 45) reported having insurance through an employer, 13% (n = 22) Medicare, 9.7% (n = 16) Marketplace coverage, and 2.6% other insurance.

Roughly half of those who transitioned to employer or marketplace coverage reported coverage gaps during the prior year. Overall, and including those who became uninsured, only 49 respondents (29.3%) who lost Medicaid transitioned to new coverage without a gap. Among respondents who had Medicaid coverage at the time of the interview, 195 (15.5%) reported a coverage gap during the prior year, potentially reflecting churn in and out of the program.

### Factors Associated With Medicaid Loss

Table 2 shows factors associated with disenrollment from Medicaid between March 2020 and fall 2023. Disenrollment was significantly higher among individuals in Arkansas than in Louisiana and Kentucky, although this difference only remained significant in Kentucky vs Arkansas after multivariate adjustment. Disenrollment was significantly higher among younger adults, rural individuals, those who were employed, and White adults (compared with non-Hispanic Black adults), although this last difference was significant only in the unadjusted analysis. Individuals receiving SNAP benefits were significantly less likely to disenroll. Moving and having an income greater than 100% of the FPL during the prior year were significant risk factors for disenrollment, while SSI was associated with lower disenrollment, but all 3 were only significant in unadjusted models. Among children (eTable 2 in Supplement 1), Arkansas had significantly higher disenrollment rates than the other states (Kentucky was excluded from this analysis, since it had 0% disenrollment by state decision in 2023), while being enrolled in SNAP was highly protective against disenrollment.

**Table 2.** Factors Associated With Adult Disenrollment From Medicaid Since March 2020^a^

	Unadjusted			Adjusted		
	Probability, %	Odds ratio (95% CI)	*P* value	Predicted probability, %	Odds ratio (95% CI)	*P* value
State						
Arkansas	16.2	1 [Reference]	1 [Reference]	14.9	1 [Reference]	1 [Reference]
Kentucky	7.0	0.39 (0.20-0.76)	.01	6.4	0.38 (0.19-0.76)	.01
Louisiana	8.1	0.46 (0.25-0.85)	.01	9.2	0.59 (0.31-1.12)	.11
Texas	14.9	0.90 (0.57-1.43)	.66	19.2	1.55 (0.87-2.78)	.14
Race and ethnicity						
Black non-Hispanic	8.2	0.55 (0.34-0.90)	.02	9.4	0.62 (0.36-1.25)	.07
Hispanic	13.4	0.96 (0.56-1.62)	.87	11.8	0.81 (0.42-1.60)	.55
White non-Hispanic	13.9	1 [Reference]	1 [Reference]	13.8	1 [Reference]	1 [Reference]
Other^b^	14.9	1.09 (0.51-2.33)	.83	16.6	1.29 (0.53-3.13)	.58
Age group, y						
19-29	16.7	1 [Reference]	1 [Reference]	14.0	1 [Reference]	1 [Reference]
30-39	12.4	0.71 (0.42-1.19)	.19	11.8	0.80 (0.45-1.43)	.45
40-49	12.8	0.73 (0.43-1.24)	.25	13.7	0.97 (0.51-1.86)	.93
50-59	13.5	0.78 (0.44-1.38)	.39	16.9	1.29 (0.64-2.57)	.48
60-64	0.6	0.03 (0.01-0.11)	<.001	0.9	0.05 (0.01-0.18)	<.001
Education						
Less than high school	9.8	1 [Reference]	1 [Reference]	12.7	1 [Reference]	1 [Reference]
High school degree	11.6	1.20 (0.64-2.23)	.57	12.1	0.93 (0.46-1.87)	.84
Some college/finished college	14.8	1.59 (0.86-2.93)	.14	13.2	1.05 (0.52-2.14)	.89
Income						
Less than 50% FPL	9.5	1 [Reference]	1 [Reference]	11.2	1 [Reference]	1 [Reference]
50%-100% FPL	12.9	1.41 (0.90-2.22)	.14	12.3	1.13 (0.65-1.96)	.67
100%-138% FPL	19.1	2.25 (1.34-3.79)	.002	16.6	1.68 (0.94-3.02)	.08
Do not know/refused	1.5	0.15 (0.02-1.11)	.06	2.3	0.17 (0.02-1.31)	.09
Employed						
No	7.5	1 [Reference]	1 [Reference]	8.7	1 [Reference]	1 [Reference]
Yes	21.0	3.29 (2.20-4.92)	<.001	17.6	2.43 (1.53-3.85)	<.001
Additional demographics						
Woman	13.0	1.14 (0.74-1.76)	.56	13.6	1.32 (0.82-2.11)	.25
Married/partnered	13.2	1.11 (0.74-1.67)	.62	12.6	0.99 (0.60-1.63)	.96
Has dependent children	11.5	0.98 (0.66-1.45)	.17	10.7	0.64 (0.40-1.02)	.06
Spanish interview	2.4	0.17 (0.02-1.31)	.09	2.4	0.14 (0.01-1.73)	.13
Rural	15.9	1.58 (1.06-2.35)	.03	17.6	2.05 (1.28-3.29)	.01
Additional factors						
Has chronic condition						
No	14.0	1 [Reference]	1 [Reference]	11.7	1 [Reference]	1 [Reference]
Yes	12.1	0.84 (0.55-1.31)	.45	13.0	1.15 (0.70-1.91)	.58
Receives SSI						
No	15.4	1 [Reference]	1 [Reference]	13.7	1 [Reference]	1 [Reference]
Yes	6.3	0.37 (0.22-0.62)	<.001	9.2	0.60 (0.35-1.04)	.07
Receives SNAP						
No	18.5	1 [Reference]	1 [Reference]	15.6	1 [Reference]	1 [Reference]
Yes	8.0	0.39 (0.22-0.62)	<.001	9.7	0.55 (0.35-1.04)	.01
Moved since March 2020						
No	9.9	1 [Reference]	1 [Reference]	10.8	1 [Reference]	1 [Reference]
Yes	15.7	1.71 (1.15-2.52)	.007	14.6	1.49 (0.94-2.36)	.09

Abbreviations: FPL, federal poverty line; SNAP, Supplemental Nutrition Assistance Program; SSI, supplemental security income.

^a^ Table reports results from a logistic regression examining characteristics associated with Medicaid loss among adult respondents; predicted probabilities were estimated using Stata (StataCorp).

^b^ Race and ethnicity were self-reported; “Other” included those who identify as American Indian or Alaskan Native, Asian, Native Hawaiian or other Pacific Islander, or who selected the Other response in the survey.

Characteristics among adults of being uninsured at time of interview after exiting Medicaid were generally similar as those for Medicaid disenrollment (regardless of coverage at the time of the interview) in unadjusted analyses, except there were no significant differences by race and ethnicity or income, and women were significantly more likely to become uninsured than men (eTable 3 in Supplement 1). In adjusted analyses, coefficients associated with state of residence, age, employment, and SSI receipt remained significant.

### Access to Care

**Figure 3.**
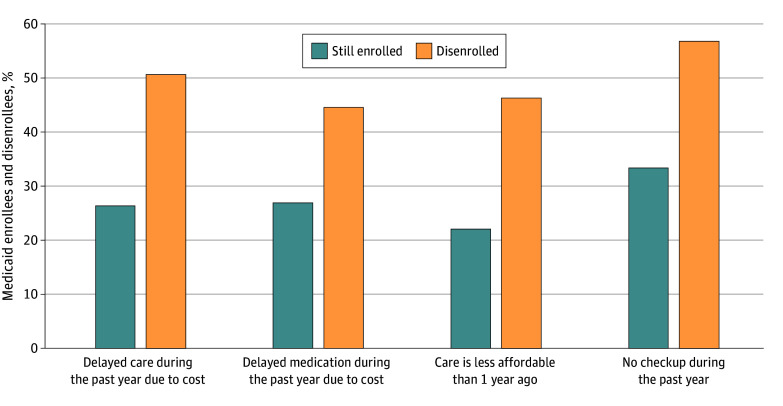
Affordability and Access to Care Among Adult Medicaid Enrollees vs Disenrollees Adjusted predicted probabilities (estimated using Stata’s “margins” command with default settings [StataCorp]) from a logistic regression using the same covariates reported in Table 2. Data were from a multimodal (telephone + internet) survey of nonelderly US citizens (aged 19-64 years) who lived in 1 of 4 Southern states (Arkansas, Kentucky, Louisiana, and Texas) and reported 2022 household incomes less than 138% of the federal poverty line and reported that they had been enrolled in Medicaid at some point since March 2020. The survey was fielded from September to November 2023. Percentages may not sum to 100 due to rounding. All reported estimates are survey-weighted.

Figure 3 shows several measures of affordability and access to care, comparing adults who remained enrolled in Medicaid with those who disenrolled from Medicaid. For all 4 measures, adults who disenrolled had significantly worse access and/or affordability, which included more cost-related delays in care (50.8% vs 26.5%), more delays or skipped doses of medications due to cost (44.8% vs 27.1%), reporting that care was less affordable than during the year before (46.5% vs 22.3%), and less likely to have had a checkup during the prior year (57.0% of those disenrolled had no checkup vs 33.6% of people who had Medicaid at time of the interview). Results were generally similar for those who became uninsured vs those with new, non-Medicaid coverage (eFigure 2 in Supplement 1).

## Discussion

In this survey of low-income households in 4 Southern states in late 2023, we find that roughly 6 months into the unwinding process, 1 in 8 Medicaid beneficiaries reported exiting the program, nearly half of these adults became uninsured, and those leaving Medicaid experienced more disruptions in medical care than those who remained enrolled. Disenrollment was highest in Arkansas, which started redeterminations earlier than the other states and conducted unwinding on an accelerated 6-month timeline. Texas had the next highest disenrollment rate, likely reflecting the fact that it was the only nonexpansion state in our sample, meaning a much smaller share of nonelderly adults in the state qualify for the program; Texas also frontloaded redeterminations for likely ineligible individuals. The lowest disenrollment rates were in Kentucky and Louisiana, which are expansion states that spread their renewals over the full year and used outside data sources to limit the burden on beneficiaries to demonstrate ongoing eligibility.^25,26^ This general pattern resembled findings from a recent analysis of administrative data for all 50 states, which found a significant association between disenrollment rates and policies, including Medicaid expansion, alternative data sources for eligibility assessment, and redetermination timing.^11^

Children in the sample were less than half as likely to lose Medicaid than adults. This may partially reflect state policy choices: Kentucky suspended redeterminations for enrollees 19 years or younger for 12 months.^17^ In addition, the income inclusion criteria for the survey, which was less than 138% of the FPL, did not capture many children enrolled in Medicaid or CHIP who may be more likely to have lost coverage than children in lower-income households. Nonetheless, because children represent nearly half of all enrollees in Medicaid and CHIP, these results suggest that millions of children are losing coverage nationally.^27^

We found that 48% of respondents who reported leaving Medicaid said they were uninsured at the time of the interview. While the remainder moved into new sources of coverage, slightly less than half of those who gained private insurance experienced a coverage gap. Prior research has found that even brief coverage gaps have been associated with disruptions in care and adverse health outcomes.^20,28,29,30,31,32^ Our survey study found higher rates of delays in care and challenges with affordability among those leaving Medicaid during unwinding that were consistent with this literature, although these findings were only correlational.^20,28,30,31,32,33^ While the unwinding process is a key area of focus in 2024, broader issues of continuity of coverage in Medicaid preceded the continuous coverage policy and will persist after the unwinding period ends.^34^ Previous research has drawn attention to the frequent disruptions in postpartum coverage in Medicaid as well as churning among children; our findings support the value of ongoing policy efforts to extend continuous eligibility provisions for these populations.^35,36,37,38^

Given the low-income nature of the survey sample, it is likely that many uninsured respondents either remained eligible for Medicaid or would qualify for substantial subsidies to purchase insurance through the Affordable Care Act marketplaces.^39^ However, fewer than 1 in 10 respondents who had lost Medicaid coverage had enrolled in a marketplace plan. This modest marketplace take-up rate was consistent with prior research and indicated that more robust outreach and assistance may be required to promote successful transitions into marketplace coverage.^40,41,42,43,44^

We identified several significant individual-level factors that were associated with Medicaid disenrollment. Younger adults, those who are working and those with higher incomes during the previous year were more likely to lose coverage (although the latter finding was no longer significant after adjustment); these factors may all reflect greater income mobility and help explain why more than a quarter of disenrollees had moved to employer coverage after Medicaid. Disenrollment was higher among rural adults and (in unadjusted analyses) among those who recently moved, which may indicate the difficulties states have reaching such enrollees to help them navigate the redetermination process. Disenrollment rates were higher for White than Black individuals (with Hispanic individuals falling in between) in unadjusted analyses. Other preliminary research on unwinding has found mixed results, with at least 1 study finding lower disenrollment among White beneficiaries; these results may vary based on the states and data sources being examined and require additional future research to assess effects on disparities.^45^

Individuals in SNAP were less likely to lose coverage, which may reflect the use of eligibility information from other programs by states to streamline redetermination, as well as greater engagement and awareness of state policies among those participating in multiple programs. SSI participation was also highly protective, although this is expected, given that SSI in these states automatically confers Medicaid coverage; the fact that there was any reported disenrollment from Medicaid among those reporting SSI may reflect respondent confusion over their SSI or Medicaid status, which is consistent with recent studies on coverage awareness during the COVID-19 pandemic.^46,47^

### Limitations

Our study had several limitations. First, our response rate was much lower than high-quality federal surveys. However, the response rate was similar to other rapid-turnaround surveys (including the US Census Bureau’s Household Pulse Survey), and previous research has validated our survey approach in terms of trends in producing similar trends in state coverage rates as the American Community Survey.^19^ This year’s survey included a partial shift to ABS, and our module on children’s coverage is new and should be considered exploratory. Our survey-reported state-level rates of Medicaid disenrollment were highly correlated with concurrent estimates from state administrative data, potentially offering reassurance for our overall approach.

Our sample was limited to residents of 4 Southern states who reported household incomes less than 138% of the FPL in 2022, which may limit generalizability.^11^ State approaches to unwinding varied considerably; thus, experiences may have been different in other states. Additionally, many individuals with higher incomes would have been affected by the Medicaid continuous coverage provision and unwinding; about half of children and nonelderly adults who had Medicaid in 2021 had household incomes greater than 138% of the FPL.^48^ Because our survey was limited to US citizens, our results also may not generalize to noncitizen permanent residents who qualify for Medicaid in those 4 states.

As with all surveys, there is potential for reporting errors. Some respondents may have been confused about their Medicaid or SSI status or misreported other characteristics or program participation. We conducted the survey in 2023 but asked about 2022 household income to establish eligibility for the survey, following previous versions of our survey, and also capture respondents whose income may have changed over time (potentially affecting Medicaid eligibility during unwinding). However, because of this, we were unable to determine directly whether respondents remained eligible for Medicaid when surveyed or would qualify for other assistance, such as marketplace subsidies. Finally, our analyses were cross-sectional and cannot establish causality.

## Conclusions

The findings of this survey study offer early evidence that approximately half of people with low incomes exiting Medicaid during unwinding have become uninsured, while the other half has largely switched to private coverage. State policy choices have been associated with significant differences in rates of coverage loss, which is consistent with the variation in our study’s state-level results. Medicaid loss was associated with greater barriers to accessing medical care. State and federal policymakers should pursue policies to mitigate adverse outcomes associated with coverage disruptions during the unwinding process and in future efforts to improve continuity of care for beneficiaries in Medicaid.

## Notes

Supplement 1. eMethods. Additional Detail on Survey Sample, Response Rates, Weighting, and Survey Question Wording eAppendix. Background on State Unwinding Policies eTable 1. Factors Associated with Disenrollment from Medicaid Since March 2020, Stratified By State eTable 2. Factors Associated with Child Disenrollment from Medicaid Since March 2020 eTable 3. Factors Associated with Losing Medicaid and Becoming Uninsured eFigure 1. Medicaid Enrollment Status at Time of Interview, Among Respondents Ever in Medicaid Since March 2020, Stratifying by Health Status, Chronic Conditions, and Disability Status eFigure 2. Affordability and Access to Care Among Adult Medicaid Enrollees versus Disenrollees, Stratified by Current Coverage Status

Supplement 2. Data sharing statement
