# Supplementary material for: Coverage and Access Changes During Medicaid Unwinding
Source: JAMA Health Forum. 2024 Jun 29;5(6):e242193. doi: 10.1001/jamahealthforum.2024.2193 (PMC11214671; doi:10.1001/jamahealthforum.2024.2193)
Supplement: Supplement 1. — eMethods. Additional Detail on Survey Sample, Response Rates, Weighting, and Survey Question Wording eAppendix. Background on State Unwinding Policies eTable 1. Factors Associated with Disenrollment from Medicaid Since March 2020, Stratified By State eTable 2. Factors Associated with Child Disenrollment from Medicaid Since March 2020 eTable 3. Factors Associated with Losing Medicaid and Becoming Uninsured eFigure 1. Medicaid Enrollment Status at Time of Interview, Among Respondents Ever in Medicaid Since March 2020, Stratifying by Health Status, Chronic Conditions, and Disability Status eFigure 2. Affordability and Access to Care Among Adult Medicaid Enrollees versus Disenrollees, Stratified by Current Coverage Status [file jamahealthforum-e242193-s001.pdf]

Supplemental Online Content

McIntyre A, Sommers BD, Aboulafia G, et al. Coverage and access changes during Medicaid unwinding. *JAMA Health Forum*. 2024;5(6):e242193. doi:10.1001/jamahealthforum.2024.2193

eMethods. Additional Detail on Survey Sample, Response Rates, Weighting, and Survey Question Wording
eAppendix. Background on State Unwinding Policies
eTable 1. Factors Associated with Disenrollment from Medicaid Since March 2020, Stratified By State
eTable 2. Factors Associated with Child Disenrollment from Medicaid Since March 2020
eTable 3. Factors Associated with Losing Medicaid and Becoming Uninsured
eFigure 1. Medicaid Enrollment Status at Time of Interview, Among Respondents Ever in Medicaid Since March 2020, Stratifying by Health Status, Chronic Conditions, and Disability Status
eFigure 2. Affordability and Access to Care Among Adult Medicaid Enrollees versus Disenrollees, Stratified by Current Coverage Status

This supplemental material has been provided by the authors to give readers additional information about their work.

© 2024 McIntyre A et al. *JAMA Health Forum.*

eMETHODS

Additional Detail on Survey Sample, Response Rates, and Weighting

Our survey was conducted by the non-partisan polling firm SSRS. The survey was fielded from September 18 through November 21, 2023. SSRS collected data online and by telephone, from 2,210 U.S. citizens age 19-64 with a household income under 138% of the federal poverty line (FPL) in Texas (n=792), Arkansas (n=727), Kentucky (n=351), and Louisiana (n=340). Our sample was designed to increase efficiency in reaching the target population. We did not collect data on individuals who did not meet this inclusion criteria.

Two main sampling frames were combined: an address-based sample (ABS) and a random digit dial (RDD) sample of landline and cell phone numbers. The RDD sample was augmented with completed surveys from a probability-based panel, and callbacks to previous respondents from previous RDD studies.

The ABS sampling frame consists of addresses in the USPS Computerized Delivery Sequence File (CDSF). This frame contains over 147 million residential addresses covering nearly 100% of all households in the U.S. An ABS frame allows for effective targeting of smaller geographic areas, in this case Census block groups (CBGs) in our four states with higher prevalence of low-income households. Households in counties where less than 3% of households are under the FPL were excluded.

Similarly, the random digit dialing (RDD) frames (both cell phone and landline) targeted likely low-income respondents by stratifying the frames based on the percent in a phone exchange's county with a household income under the FPL.

A portion of each cell phone and landline stratum consisted of directory-listed low-income households (LHH), identified through commercial listings by their annual income. SSRS obtained an estimate of the number of these listed households within each of the income-based strata and generated estimates of the low-income population in each stratum. The LHH and lower income strata were oversampled.

A small portion of respondents were reached by calling back respondents who completed an interview on the SSRS Omnibus poll or other RDD polls in recent years and whose responses indicated they might meet the current study's eligibility criteria based on age and income (less than $30,000 annually). From this sample, 85 respondents were reached (26 in Arkansas, 15 in Kentucky, 14 in Louisiana and 30 in Texas) – referred to below as the "Callback" sample.

An additional 40 respondents (32 in Texas and 8 in Kentucky) were reached through the SSRS Opinion Panel, a nationally representative probability-based web panel. Members were probabilistically recruited in one of two ways: (1) through invitations mailed to respondents randomly sampled from an Address-Based Sample (ABS), or (2) from a dual-frame random digit dial (RDD) sample, through the SSRS Omnibus survey

© 2024 McIntyre A et al. *JAMA Health Forum.*

platform. Panelists who had previously indicated they might meet the eligibility criteria were invited to participate. 30 completed the survey online and 10 who do not use the internet were reached by phone.

Response rates were calculated using AAPOR's RR3 formula. The overall response rate for the ABS and telephone sample was 5%. Below are response rates for each sample type by state.

**ABS**	
Arkansas	8%
Kentucky	8%
Louisiana	6%
Texas	6%
**RDD**	
Arkansas Landline	2%
Arkansas Cell Phone	3%
Arkansas Callback	21%
Kentucky Landline	2%
Kentucky Cell Phone	3%
Kentucky Callback	10%
Louisiana Landline	1%
Louisiana Cell Phone	2%
Louisiana Callback	8%
Texas Landline	1%
Texas Cell Phone	2%
Texas Callback	5%

We used survey weights to address potential non-response bias and generate estimates representative of the target population in each of the study states. The sample was weighted in stages. The first stage of the weighting was the application of a base weight to account for differing selection probabilities and response rates. In the second stage sample demographics were post-stratified to match population parameters. The sample was weighted to match population estimates in each state for the 19–64-year-old citizen population with household incomes under 138% FPL, based on the 2021 ACS. The weighting parameters were age by sex, race/ethnicity, education, marital status, household tenure, and geographic region within-state. Additional parameters for Arkansas were age by education and age by race.

Item Non-Response

For item non-response, we followed the basic approach of our prior research with similar data. We omitted from each regression any observation with a missing value for the outcome in question (which ranged from 0.8 to 1.0%). For individual-level covariates, non-response for race/ethnicity (0.6%) was treated as "other." For 0.4% of

© 2024 McIntyre A et al. *JAMA Health Forum.*

the weighted sample with missing age, 0.4% with missing education, and 0.03% with missing marital status, we imputed these values based on multivariate regression models using race/ethnicity, gender, income, family size, urban/rural location, cell phone usage, and political affiliation as predictor variables. Our overall results were extremely similar if we omitted observations with imputed covariates (0.6% of the sample) from the analysis.

Coverage Definition

Insurance type was categorized into mutually-exclusive categories using the following hierarchy: Medicaid, Medicare, Marketplace, Employer-Sponsored Insurance (ESI), and other insurance.

Regression Equations

1) Predictors of disenrollment from Medicaid – Table 2, adjusted model (sample limited to adults who reported having Medicaid at any point since March 2020):

$$EverMedicaidDisenrolled_i = \beta_1 + \beta_1 State_i + \beta_2 RaceEthnicity\ _i + \beta_3 AgeGroup_i + \beta_4 Education + \beta_5 Employment\_status + X_i + \varepsilon_i \quad \textbf{(1)}$$

where i indexed individuals. $EverMedicaidDisenrolled_i$ was a binary outcome indicating whether an individual still had their Medicaid coverage, X_i was a vector of additional demographics and other factors (gender, marital status, whether the respondent has a dependent, whether the interview was conducted in Spanish, urban vs. rural status, SSI receipt, SNAP receipt, and whether the respondent has moved since March 2020). We also estimated unadjusted associations by regressing our outcome of interest on each covariate individually.

2) Predictors of current insurance status – Appendix Table 1, adjusted model (sample limited to adults who reported having Medicaid at any point since March 2020):

$$Uninsured_i = \beta_1 + \beta_1 State_i + \beta_2 RaceEthnicity\ _i + \beta_3 AgeGroup_i + \beta_4 Education + \beta_5 Employment\_status + X_i + \varepsilon_i \quad \textbf{(2)}$$

where i indexed individuals. $Uninsured_i$ was a binary outcome indicating whether an individual was uninsured at time of interview. X_i was a vector of additional demographics and other factors (gender, marital status, whether the respondent has a dependent, whether the interview was conducted in Spanish, urban vs. rural status, SSI receipt, SNAP receipt, and whether the respondent has moved since March 2020). We specified the unadjusted predictive models by regressing our outcome of interest on each covariate individually.

© 2024 McIntyre A et al. *JAMA Health Forum.*

SURVEY QUESTIONS

The following survey items were used for our study outcomes:

<u>Health Insurance</u>

Current coverage status – respondent

Ask all

1) Next are some questions about the health insurance that you, yourself, currently have. Do you currently have this type of health insurance, or not?
   a. [State Medicaid Plan Name]? This is a Medical Assistance or government-assistance plan for those with low incomes or a disability. [IF STATE=KY: It is also sometimes called "Aetna Better Health," “Passport by Molina,” or “Wellcare.”]
   b. Medicare? The government program for people 65 and older, or people with certain disabilities.
   c. a military health care plan, such as TRI-CARE, CHAMPUS, or CHAMP-VA?
   d. a health plan you got through an employer or union? This also includes through a spouse’s or family member’s employer or union.
   e. a health insurance plan that you signed up for through [State Marketplace Name] or a health insurance Marketplace created by the national health reform law? The national health reform law is sometimes referred to as Obamacare or the Affordable Care Act.
   f. a health plan that you bought directly from an insurance company, not through an employer or union, and not through a health insurance Marketplace?
   g. some other kind of health insurance not already mentioned?

If no to all

   h. Does this mean you have no health insurance of any kind?

Ask if currently insured

2) Out of the past 12 months, how many months did you have health insurance?
   a. You had insurance for the whole year (12 months)
   b. You had insurance for 8-11 months
   c. You had insurance for 4-7 months
   d. You had insurance for 3 months or less

Ask if currently uninsured

3) You told us that you currently do not have any health insurance. Out of the past 12 months, how many months did you have health insurance?
   a. You had insurance for 8-11 months

b. You had insurance for 4-7 months
c. You had insurance for 1-3 months
d. You had insurance for 0 months or never had insurance over the past year

Current coverage status – dependent

Ask if respondent reports being parent or guardian for at least one child 18 or younger

4) If respondent has one child:
You previously mentioned that you are the parent or guardian for a child age 18 or under in your household. I'm now going to ask you about their health insurance.

If respondent has more than one child:

5) You previously mentioned that you are the parent or guardian for more than one child age 18 of younger in your household. For the following questions, I'm going to ask you to focus on the child who had the most recent birthday.

For each type of health insurance, please indicate 'yes' if your child has it and 'no' if they do not. You can answer 'yes' more than once.

a. [State Medicaid Plan Name] or [STATE CHIP NAME]? [IF STATE=KY: It is also sometimes called "Aetna Better Health," "Humana/ CareSource," "Passport," or "Wellcare."]
b. Medicare?
c. a military health care plan, such as TRI-CARE, CHAMPUS, or CHAMP-VA?
d. a health plan you got through an employer or union?
e. a health insurance plan that you signed up for through [State Marketplace Name] or a health insurance Marketplace created by the national health reform law?
f. a health plan that you bought directly from an insurance company, not through an employer or union, and not through a health insurance Marketplace?
g. some other kind of health insurance not already mentioned?

If no to all

h. Does this mean your child does not have health insurance of any kind?

Experience with Medicaid coverage – respondent

Ask those who do not currently have Medicaid

6) Now, thinking back to the start of the COVID-19 pandemic, in March of 2020. At any point since that time, did you yourself have health insurance through [INSERT MEDICAID NAME]?

Ask if ever had Medicaid since March 2020 (but does not currently have it)

© 2024 McIntyre A et al. *JAMA Health Forum.*

7) What is the main reason you no longer have Medicaid?

a. You gained new coverage and chose to drop Medicaid
b. You moved to a new state
c. You no longer qualify for Medicaid
d. You tried to stay in Medicaid, but[IF WEB: I/IF CATI: you] could not complete the renewal process
e. Some other reason [Please Specify]

8) About when did your Medicaid Coverage end? Would you say it was…?

a. Before January 2023
b. Between January and April 2023
c. After April 2023

Health Care Access and Utilization

Ask all

9) At any time in the last year, have you waited to seek medical care or chosen not to seek medical care for an illness, injury, or condition because you couldn't afford it?

10) At any time in the last year, have you ended up taking less medication than was prescribed for you because you couldn't afford it? This includes skipping doses, or taking a smaller dose than was prescribed, or not filling a prescription right away.

11) Thinking about the past 12 months, would you say the cost of your health care has become more affordable, less affordable, or has it stayed about the same?

a. Much more affordable
b. Slightly more affordable
c. About the same
d. Slightly less affordable
e. Much less affordable

12) Have you had a routine checkup in the last 12 months, or not? A routine checkup is a general physical exam, not an exam for a specific injury, illness, or condition.

Current Employment

Ask all

13) Do you currently work for pay at a job or business?

Coverage Loss Risk Factors

Ask all

© 2024 McIntyre A et al. *JAMA Health Forum.*

14) Thinking back to the start of the COVID-19 pandemic, which was in March of 2020. How many times have you moved or changed residences since that time?

a. Zero times, you lived in the same house or apartment the entire time
b. One time
c. Two times

15) The next question asks about public benefits you or your family may have received in the past 12 months. Did you or anyone in your household receive the following benefits at any time since [CURRENT MONTH] [CURRENT YEAR-1]?

16) Please include benefits received by you, your spouse or partner, if applicable, and any of your children or stepchildren under 19 who are living with you.

a. SNAP, the Supplemental Nutrition Assistance Program, formerly known as the Food Stamp Program.
b. WIC, the Women, Infants, and Children Program
c. SSI, Supplemental Security Income, which provides cash assistance to low-income aged, blind and disabled persons

© 2024 McIntyre A et al. *JAMA Health Forum.*

eAppendix. Background on State Unwinding Policies

Some state differences in our survey may be attributable to varied policy contexts, including when states had initiated renewals, how many months they planned to use for the redetermination process, whether they prioritized enrollees who were likely to be ineligible for Medicaid based on the state's data, and how many flexibilities they sought from the federal government to support their renewal processes. Below we have detailed some of these differences for our states.

	Month renewals initiated[1]	First month of procedural terminations[1]	Estimated time to complete all renewals[1]	State prioritized "likely ineligible" enrollees for redetermination[1]	Ex parte renewals conducted during PHE[2]	Pre-populated renewal forms sent if unable to process ex parte[2]
**AR**	February 2023	April 2023	< 9 months	Yes	x	x
**KY**	April 2023	June 2023	12-14 months	No	x	
**LA**	April 2023	July 2023	12-14 months	No	x	
**TX**	April 2023	June 2023	12-14 months	Yes	x	x

All states except Arkansas anticipated taking at least one year to conduct their redeterminations. Terminations in Arkansas were conducted over six months, as noted in the manuscript.

All four states continued conducting ex parte renewal processes during the federal public health emergency (PHE), though enrollees remained enrollees regardless of the outcome of these renewals. In Arkansas and Texas, renewal paperwork was sent in cases where ex parte renewal failed, though enrollees retained coverage regardless of whether they returned that paperwork while continuous coverage was in effect

States also varied in the extent to which they took advantage of federal flexibilities using 1902(e)(14)(A) waivers to support their redetermination and renewal processes. The table on the next page details which strategies each of the four states included in our survey pursued.

Our state-level estimates of adult coverage loss were strongly correlated with administrative records of coverage loss in late 2023 (rho=0.92). Examining cumulative Medicaid terminations through September 2023 as a share of March 2023 enrollment, Arkansas had disenrolled 25.5%, Kentucky had terminated 7.3%, Louisiana had terminated 8.6%, and Texas had terminated 15.6% of enrollees. Our survey estimates for Medicaid loss rates were 16.2% in Arkansas, 7.0% in Kentucky, 8.2% in Louisiana, and 14.9% in Texas.

---

[1] State Approaches to the Unwinding Period [Internet]. KFF; 2023 Jan [cited 2024 May 2]. Available from: https://www.kff.org/other/state-indicator/state-approaches-to-the-unwinding-period/

[2] Brooks T, Gardner A, Osorio A, Yee P, Tolbert J, Corallo B, Moreno S, Ammula M, et al. Medicaid and CHIP Eligibility, Enrollment, and Renewal Policies as States Prepare for the Unwinding of the Pandemic-Era Continuous Enrollment Provision [Internet]. KFF; 2022 Mar. Available from: https://www.kff.org/medicaid/report/medicaid-and-chip-eligibility-enrollment-and-renewal-policies-as-states-prepare-for-the-unwinding-of-the-pandemic-era-continuous-enrollment-provision/

© 2024 McIntyre A et al. *JAMA Health Forum.*

State strategies to support Medicaid renewals using 1902(e)(14)(A) waivers

	AR	KY	LA	TX
**Increase Ex Parte Renewal Rates**				
Enroll and/or Renew Individuals Based on SNAP Eligibility (MAGI)	x	x	x	x
Enroll and/or Renew Individuals Based on SNAP Eligibility (Non-MAGI)	x			
Enroll and/or Renew Individuals Based on TANF Eligibility (MAGI)			x	
Enroll and/or Renew Individuals Based on TANF Eligibility (Non-MAGI)				
Renew Medicaid Eligibility for Individuals with No Income and No Data Returned on an Ex Parte Basis		x	x	
Renew Medicaid Eligibility for Individuals with Income at or below 100% of Federal Poverty Level (FPL) and No Data Returned		x		
Renew Medicaid Eligibility for Individuals for Whom Information from the Asset Verification System (AVS) Is Not Returned Within a Reasonable Timeframe		x	x	
Renew Medicaid Eligibility for Individuals with Only Title II or Other Stable Sources of Income Without Checking Required Data Sources				
Renew Medicaid Eligibility Based on a Simplified Asset Verification Process		x		
Suspend the Requirement to Apply for Other Benefits Under 42 C.F.R. 435.608		x		
Suspend the Requirement to Cooperate with the Agency in Establishing the Identity of a Child's Parents and in Obtaining Medical Support		x		
Ex Parte Attempt Prior to Termination				
Other Ex Parte Strategies		x		
**Support Enrollees with Renewal Form Submission or Completion to Reduce Procedural Terminations**				
Permit Managed Care Plans to Provide Assistance to Enrollees to Complete and Submit Medicaid Renewal Forms	x	x		x
Permit the Designation of an Authorized Representative for the Purposes of Signing an Application or Renewal Form via Telephone without a Signed Designation				
Waive the Recording of the Telephone Signature from the Applicant or Beneficiary		x		
Use a Simplified Renewal Form, Only Asking if an Individual's Income and Assets (if applicable) Remain Below the Eligibility Standard				
**Update Contact Information**				
Partner with Managed Care Plans to Update In-State Beneficiary Contact Information	x	x	x	x
Partner with National Change of Address Database and/or United States Postal Service Forwarding Address to Update In-State Beneficiary Contact Information	x		x	x
Partner with Enrollment Brokers to Update In-State Beneficiary Contact Information			x	
Partner with PACE Organizations to Update In-State Beneficiary Contact Information			x	
Other Contact Information Strategy	x			
**Facilitate Reinstatement of Eligible Individuals for Procedural Reasons**				
Designate the State Agency as a Qualified Entity to Make Determinations of Presumptive Eligibility on a MAGI Basis for Individuals Disenrolled from Medicaid or CHIP				
Designate Pharmacies, CBOs, or Others as a Qualified Entity to Make Determinations of Presumptive Eligibility on a MAGI Basis for Individuals Disenrolled from Medicaid or CHIP				
Reinstate Eligibility Effective on the Individual's Termination Date for those Procedurally Disenrolled and Subsequently Redetermined Eligible During the Reconsideration Period		x		
Extend Automatic Reenrollment into a Medicaid Managed Care Plan up to 120 Days After a Loss of Medicaid Coverage		x		
Other				
**Other Strategies**				
Extend Timeframe to Take Final Administrative Action on Fair Hearing Requests		x	x	
Other Strategies Related to Fair Hearings				
Delay Resumption of Medicaid Premiums Imposed Under the State Plan Until After a Redetermination of Eligibility				
**Total Strategies Used**	**6**	**14**	**9**	**4**

Notes: *Data are from the Centers for Medicare and Medicaid Services (https://www.medicaid.gov/resources-for-states/coronavirus-disease-2019-covid-19/unwinding-and-returning-regular-operations-after-covid-19/covid-19-phe-unwinding-section-1902e14a-waiver-approvals/index.html) as of May 1, 2024. The "other" contact information strategy used by Arkansas was accepting updated contact information from a Qualified Health Plan (QHP). The "other" ex parte strategy used by Kentucky was suspending renewals for children under age 19.*

© 2024 McIntyre A et al. *JAMA Health Forum.*

eTable 1. Factors Associated with Disenrollment from Medicaid Since March 2020, Stratified By State

	Arkansas			Kentucky			Louisiana			Texas		
	Predicted Probability	Odds Ratio	p-value	Predicted Probability	Odds Ratio	p-value	Predicted Probability	Odds Ratio	p-value	Predicted Probability	Odds Ratio	p-value
**Race/Ethnicity**												
NH White	18.7%	1.00	Ref	8.0%	1.00	Ref	8.7%	1.00	Ref	18.3%	1.00	Ref
NH Black	11.4%	0.51	0.090		N/A		5.6%	0.53	0.381	17.2%	0.92	0.868
Hispanic	8.9%	0.37	0.095		N/A		12.0%	1.67	0.652	17.4%	0.94	0.882
Other	20.2%	1.13	0.865	18.8%	5.39	0.097	18.1%	3.55	0.177	8.2%	0.37	0.453
**Age Group**												
19-29	22.2%	1.00	Ref	10.6%	1.00	Ref	6.7%	1.00	Ref	15.6%	1.00	Ref
30-39	16.0%	0.62	0.309	2.4%	0.11	0.193	6.6%	0.97	0.977	19.3%	1.33	0.581
40-49	14.3%	0.54	0.214	4.7%	0.27	0.260	15.1%	3.81	0.109	20.5%	1.44	0.488
50-59	18.0%	0.74	0.539	21.7%	4.01	0.184	11.4%	2.29	0.271	13.5%	0.84	0.821
60-64	0.7%	0.02	0.001	1.8%	0.08	0.083	3.1%	0.35	0.351		N/A	
**Education**												
Less than high school	15.2%	1.00	Ref	11.1%	1.00	Ref	11.7%	1.00	Ref	18.7%	1.00	Ref
High school degree	18.1%	1.28	0.674	5.3%	0.30	0.183	6.7%	0.40	0.326	14.8%	0.73	0.546
Some college/finished degree	15.1%	0.99	0.985	11.7%	1.11	0.907	8.7%	0.60	0.490	18.6%	0.99	0.984
**Income Level**												
Under 50% FPL	12.4%	1.00	Ref	6.9%	1.00	Ref	3.7%	1.00	Ref	20.5%	1.00	Ref
50 - 100% FPL	15.3%	1.31	0.533	12.4%	2.70	0.179	10.5%	4.44	0.053	12.8%	0.54	0.246
100-138% FPL	24.7%	2.64	0.038	4.6%	0.54	0.652	13.4%	6.70	0.043	21.2%	1.04	0.939
Don't know/refused	9.6%	0.72	0.753		N/A			N/A			N/A	
**Employed**												
No	10.0%	1.00	Ref	5.7%	1.00	Ref	6.4%	1.00	Ref	12.2%	1.00	Ref
Yes	24.6%	3.29	0.002	17.3%	8.28	0.064	9.7%	1.90	0.515	25.0%	2.55	0.014
**Additional demographics**												
Female	18.4%	1.63	0.213	6.7%	0.43	0.296	8.0%	0.83	0.809	19.3%	1.74	0.202
Married/partnered	13.2%	0.60	0.220	8.3%	0.90	0.869	12.3%	2.77	0.104	19.4%	1.31	0.563

© 2024 McIntyre A et al. *JAMA Health Forum.*

Has dependent children	16.3%	0.97	0.933	8.0%	0.84	0.882	5.5%	0.35	0.160	12.9%	0.40	0.045
SpanishInterview	N/A			N/A			N/A			2.8%	0.12	0.101
Rural	19.0%	1.52	0.225	14.2%	61.92	0.002	11.1%	1.85	0.255	25.0%	1.86	0.218
**Additional Factors**												
**Has Chronic Condition**												
No	16.5%	1.00	Ref	5.1%	1.00	Ref	4.7%	1.00	Ref	18.8%	1.00	Ref
Yes	16.5%	0.99	0.987	9.2%	2.70	0.328	10.5%	3.48	0.256	16.5%	0.84	0.672
**Receives SSI**												
No	17.7%	1.00	Ref	9.3%	1.00	Ref	10.5%	1.00	Ref	17.3%	1.00	Ref
Yes	12.6%	0.63	0.249	6.6%	0.56	0.573	0.9%	0.05	0.012	16.5%	0.94	0.901
**Receives SNAP**												
No	18.4%	1.00	Ref	16.1%	1.00	Ref	11.4%	1.00	Ref	19.8%	1.00	Ref
Yes	13.6%	0.66	0.270	4.2%	0.11	0.011	4.4%	0.25	0.051	15.4%	0.71	0.444
**Moved since March 2020**												
No	17.3%	1.00	Ref	7.4%	1.00	Ref	5.0%	1.00	Ref	12.7%	1.00	Ref
Yes	15.7%	0.87	0.685	10.0%	1.75	0.484	11.4%	3.41	0.120	21.6%	2.01	0.102

NOTES: : Table reports results from a logistic regression examining predictors of Medicaid loss among adult respondents; predicted probabilities were estimated using the Stata's "margins" command with default settings. Data are from a multimodal (telephone + internet) survey of nonelderly U.S. citizens (aged 19-64) who lived in one of four Southern states (AR, KY, LA, and TX) and reported 2022 household below 138% of the federal poverty line (FPL) and reported that they had been enrolled in Medicaid at some point since March 2020. The survey was fielded from September to November 2023. All reported estimates are survey-weighted. N/A indicates the level was omitted from the model for perfectly predicting the outcome (ie, everyone with the covariate level had the same outcome) - this causes those observations to be excluded from the model. N/A indicates the variable was omitted for collinearity, this does not drop any observations from the model.

eTable 2. Factors Associated with Child Disenrollment from Medicaid Since March 2020

	Adjusted		
**State**	**Predicted probability**	**Odds ratio**	**p-value**
Arkansas	11.1%	1.00	Ref
Texas	4.5%	0.35	0.04
Louisiana	0.8%	0.05	0.01
**Race/Ethnicity**			
NH White	5.1%	1.00	Ref
NH Black	7.2%	1.52	0.44
Hispanic	7.2%	1.51	0.49
Other	10.0%	2.29	0.16
**Education**			
Less than high school	7.7%	1.00	Ref
High school degree	5.6%	0.69	0.59
Some college/finished college	6.9%	0.87	0.84
**Income Level**			
Under 50% FPL	4.1%	1.00	Ref
50 - 100% FPL	5.4%	1.37	0.55
100-138% FPL	9.2%	2.54	0.06
Don't know/refused	20.0%	7.28	0.05
**Employed**			
No	6.7%	1.00	Ref
Yes	6.1%	0.89	0.77
**Additional demographics**			
Married/partnered	6.2%	0.91	0.83
SpanishInterview	6.6%	1.04	0.97
Rural	3.4%	0.34	0.06
**Additional factors**			
Receives SSI			
No	6.6%	1.00	Ref
Yes	5.1%	0.73	0.51
Receives SNAP			
No	9.7%	1.00	Ref
Yes	3.1%	0.28	0.01
Moved since March 2020			

No	6.5%	1.00	Ref
Yes	6.3%	0.96	0.92

NOTES: Table reports results from a logistic regression examining predictors of Medicaid loss among children, for respondents who reported that they had a dependent child who had been enrolled in Medicaid at some point since March 2020. Predicted probabilities were estimated using the Stata's "margins" command with default settings. Data are from a multimodal (telephone + internet) survey of nonelderly U.S. citizens (aged 19-64) who lived in one of four Southern states (AR, KY, LA, and TX) and reported 2022 household below 138% of the federal poverty line (FPL). The survey was fielded from September to November 2023. All reported estimates are survey-weighted.

eTable 3. Factors Associated with Losing Medicaid and Becoming Uninsured

	Unadjusted			Adjusted		
	Probability	Odds ratio	p-value	Predicted probability	Odds ratio	p-value
**State**						
Arkansas	8.3%	1.00	Ref	8.0%	1.00	Ref
Kentucky	2.3%	0.26	0.01	2.4%	0.26	0.02
Texas	6.9%	0.82	0.54	7.4%	0.91	0.80
Louisiana	3.1%	0.35	0.02	3.2%	0.36	0.02
**Race/Ethnicity**						
NH White	6.2%	1.00	Ref	6.5%	1.00	Ref
NH Black	4.4%	0.69	0.26	4.6%	0.68	0.28
Hispanic	6.0%	0.96	0.91	4.8%	0.71	0.44
Other	6.7%	1.09	0.88	7.9%	1.25	0.68
**Age Group**						
19-29	8.6%	1.00	Ref	7.0%	1.00	Ref
30-39	6.7%	0.77	0.45	6.0%	0.83	0.63
40-49	6.9%	0.79	0.48	7.3%	1.05	0.90
50-59	3.0%	0.33	0.03	4.1%	0.55	0.28
60-64	0.2%	0.02	0.00	0.3%	0.03	0.00
**Education**						
Less than high school	4.0%	1.00	Ref	6.0%	1.00	Ref
High school degree	6.2%	1.57	0.33	6.6%	1.12	0.83
Some college/finished college	6.1%	1.53	0.36	5.1%	0.83	0.73
**Income Level**						
Under 50% FPL	5.1%	1.00	Ref	6.1%	1.00	Ref
50 - 100% FPL	6.2%	1.21	0.54	5.6%	0.90	0.78
100-138% FPL	6.7%	1.32	0.47	6.0%	0.97	0.95
Don't know/refused	1.5%	0.28	0.224	3.7%	0.57	0.605
**Employed**						
No	3.4%	1.00	Ref	4.0%	1.00	Ref
Yes	9.8%	3.06	0.00	8.0%	2.17	0.02
**Additional demographics**						
Female	7.2%	2.50	0.02	7.3%	2.57	0.02
Married/partnered	6.3%	1.16	0.62	6.2%	1.13	0.72

Has dependent children	7.1%	1.58	0.11	5.4%	0.80	0.50
SpanishInterview	2.4%	0.39	0.37	2.4%	0.37	0.42
Rural	5.8%	1.01	0.96	6.1%	1.08	0.80
**Additional factors**						
Has Chronic Condition*						
No	5.5%	1.00	Ref	4.6%	1.00	Ref
Yes	5.8%	1.06	0.86	6.3%	1.44	0.28
Receives SSI						
No	7.4%	1.00	Ref	6.7%	1.00	Ref
Yes	2.1%	0.27	0.00	3.0%	0.42	0.05
Receives SNAP						
No	7.3%	1.00	Ref	6.4%	1.00	Ref
Yes	4.6%	0.62	0.08	5.3%	0.81	0.55
Moved since March 2020						
No	3.9%	1.00	Ref	4.6%	1.00	Ref
Yes	8.1%	2.18	0.01	6.9%	1.58	0.16

NOTES: Table reports results from a logistic regression examining predictors of losing Medicaid and becoming uninsured among adult respondents; predicted probabilities were estimated using the Stata's "margins" command with default settings. Data are from a multimodal (telephone + internet) survey of nonelderly U.S. citizens (aged 19-64) with incomes below 138% of the federal poverty line (FPL) who reported that they had been enrolled in Medicaid at some point since March 2020. The survey was fielded from September to November 2023. Percentages may not sum to 100 due to rounding. All reported estimates are survey-weighted.

eFigure 1. Medicaid Enrollment Status at Time of Interview, Among Respondents Ever in Medicaid Since March 2020, Stratifying by Health Status, Chronic Conditions, and Disability Status

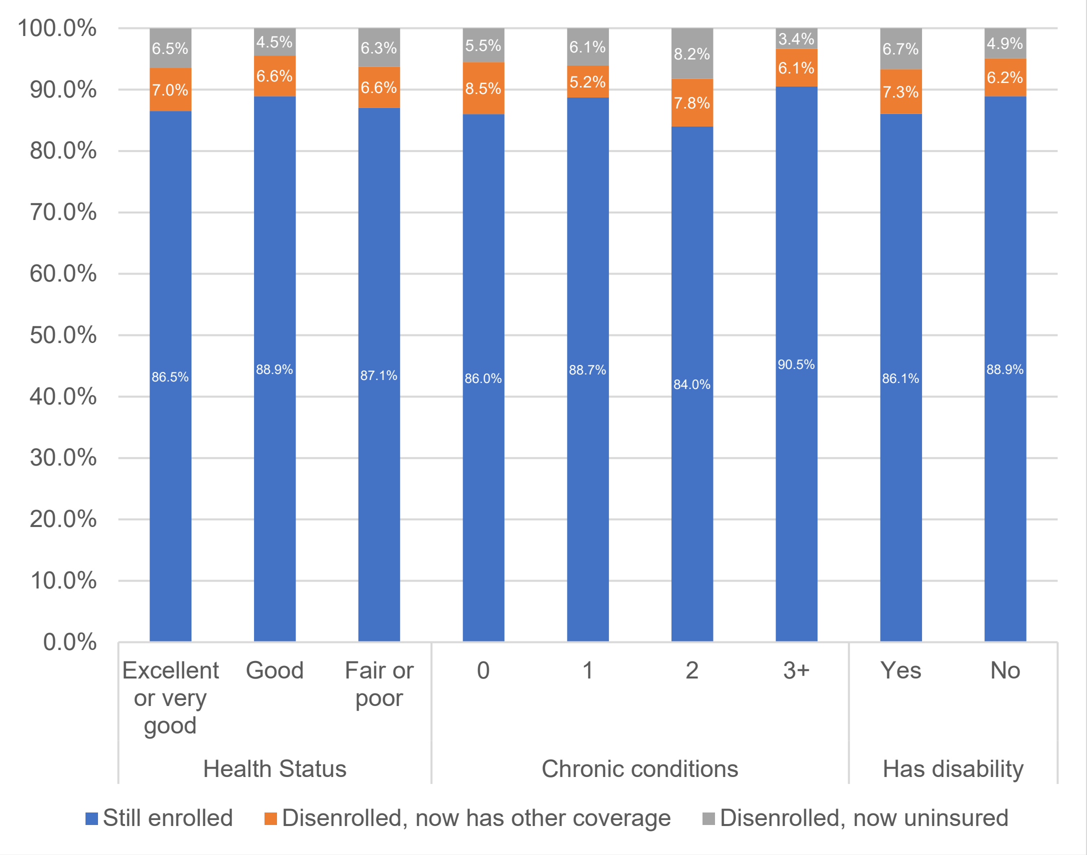

NOTES: Figure reports survey-weighted rates of coverage at time of interview among people who reported having Medicaid at any time since March 2020. Data are from a multimodal (telephone + internet) survey of nonelderly U.S. citizens (aged 19-64) with incomes below 138% of the federal poverty line (FPL) who reported that they had been enrolled in Medicaid at some point since March 2020. The survey was fielded from September to November 2023. Percentages may not sum to 100 due to rounding. All reported estimates are survey-weighted.

eFigure 2. Affordability and Access to Care Among Adult Medicaid Enrollees versus Disenrollees, Stratified by Current Coverage Status

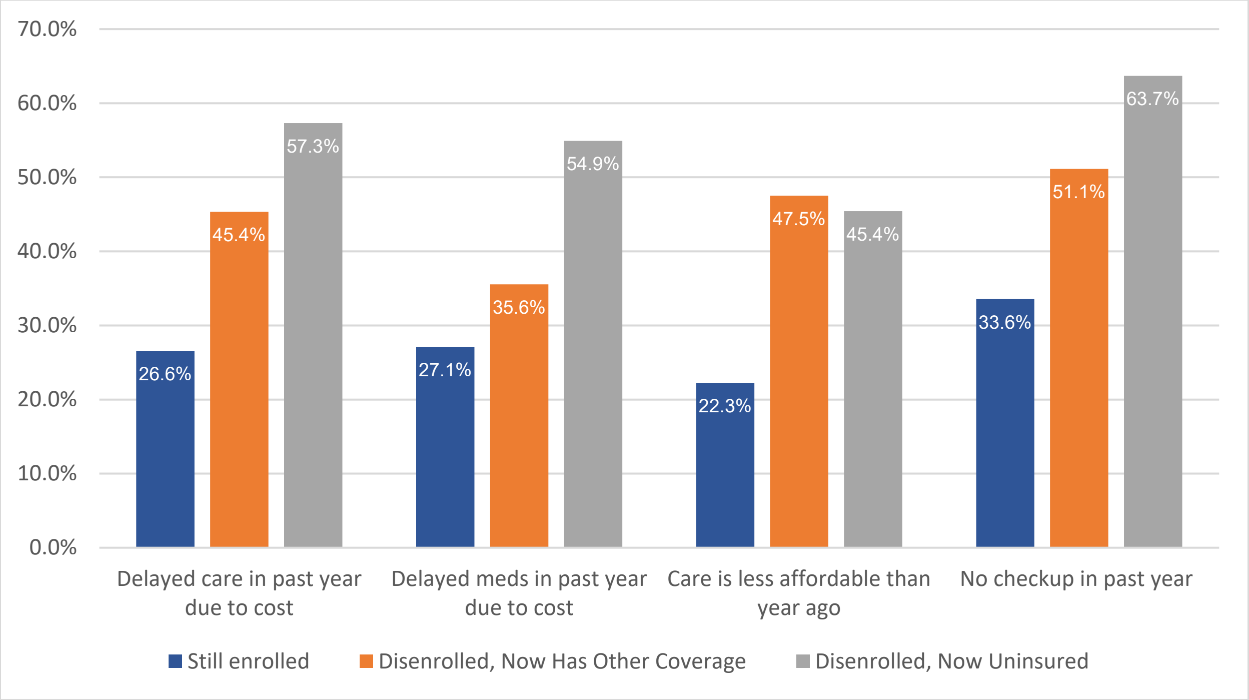

NOTES: Figure reports adjusted predicted probabilities (estimated using Stata's "margins" command with default settings) from a logistic regression using the same covariates reported in Table 2. Data are from a multimodal (telephone + internet) survey of nonelderly U.S. citizens (aged 19-64) who lived in one of four Southern states (AR, KY, LA, and TX) and reported 2022 household incomes below 138% of the federal poverty line (FPL) and reported that they had been enrolled in Medicaid at some point since March 2020. The survey was fielded from September to November 2023. Percentages may not sum to 100 due to rounding. All reported estimates are survey-weighted.
