# Supplementary material for: Coverage and Access Changes During Medicaid Unwinding
Source: JAMA Health Forum. 2024 Jun 29;5(6):e242193. doi: 10.1001/jamahealthforum.2024.2193 (PMC11214671; doi:10.1001/jamahealthforum.2024.2193)
Supplement: Supplement 2. — Data sharing statement [file jamahealthforum-e242193-s002.pdf]

Data Sharing Statement

McIntyre. Coverage and Access Changes During Medicaid Unwinding. *JAMA Health Forum*. Published June 29, 2024. doi:10.1001/jamahealthforum.2024.2193

Data
Data available: No
